# A Multicenter Analysis of Elvitegravir Use During Pregnancy on HIV Viral Suppression and Perinatal Outcomes

**DOI:** 10.1093/ofid/ofz129

**Published:** 2019-03-18

**Authors:** Martina L Badell, Anandi N Sheth, Florence Momplaisir, Lisa Rahangdale, JoNell Potter, Padmashree C Woodham, Gweneth B Lazenby, William R Short, Scott E Gillespie, Nevert Baldreldin, Emily S Miller, Gregg Alleyne, Lunthita M Duthely, Stephanie M Allen, Judy Levison, Rana Chakraborty, HOPES (HIV and OB Pregnancy Education Study) Group

**Affiliations:** 1 Division of Maternal-Fetal Medicine, Department of Gynecology and Obstetrics, Emory University School of Medicine, Atlanta, Georgia; 2 Division of Infectious Diseases, Department of Medicine, Emory University School of Medicine, Atlanta, Georgia; 3 Division of Infectious Diseases and HIV Medicine, Drexel University School of Medicine, Philadelphia, Pennsylvania; 4 Department of Obstetrics & Gynecology, University of North Carolina, Chapel Hill, North Carolina; 5 Department of Obstetrics and Gynecology, University of Miami Miller School of Medicine, Miami, Florida; 6 Division of Maternal-Fetal Medicine, Department of Obstetrics and Gynecology, Mercer University School of Medicine at the Medical Center Navicent Health, Macon, Georgia; 7 Departments of Obstetrics and Gynecology and Medicine, Medical University of South Carolina, Charleston, South Carolina; 8 Division of Infectious Diseases, Perelman School of Medicine, University of Pennsylvania, Philadelphia, Pennsylvania; 9 Department of Pediatrics, Emory University School of Medicine, Atlanta, Georgia; 10 Division of Maternal Fetal Medicine, Department of Obstetrics and Gynecology, Northwestern University Feinberg School of Medicine, Chicago, Illinois; 12 Department of Obstetrics and Gynecology, Baylor College of Medicine, Houston, Texas; 13 Department of Pediatrics and Adolescent Medicine, Mayo Clinic College of Medicine, Rochester, Minnesota

**Keywords:** HIV viral suppression, obstetrics and gynecology, perinatal outcomes, prevention of mother-to-child transmission

## Abstract

**Background:**

There is a knowledge gap on the clinical use of elvitegravir (EVG) during pregnancy and maternal viral suppression. Our objective was to evaluate the effects of EVG use in pregnancy on rates of HIV virologic suppression and perinatal outcomes.

**Methods:**

We conducted a retrospective, multicenter study of pregnant women living with HIV (WLHIV) who used EVG-containing antiretroviral therapy (ART) between January 2014 and March 2017 at 9 tertiary care centers in the United States. WLHIV were included if they took EVG at any time during pregnancy. We described the characteristics of the WLHIV using EVG during the study period and evaluated the rates of HIV suppression and perinatal outcomes.

**Results:**

Among 134 pregnant WLHIV who received EVG at any time during pregnancy, viral suppression at delivery (HIV-1 RNA < 40 copies/mL) occurred in 81.3%. In WLHIV who initiated EVG before pregnancy and continued through delivery (n = 68), the rate of viral suppression at delivery was 88.2%. The average gestational age at the time of delivery was 37 weeks 6 days, and the overall rate of preterm birth was 20%. No cases of open neural tube defects were noted in women on EVG at the time of conception (n = 82). The perinatal HIV transmission rate was 0.8%.

**Conclusions:**

EVG use was associated with high sustained levels of HIV suppression during pregnancy and a low rate of perinatal HIV transmission.

The Panel on Treatment of Pregnant Women with HIV Infection and Prevention of Perinatal Transmission recommends antiretroviral therapy (ART) during pregnancy for women living with HIV (WLHIV) [1]. Prompt initiation and adherence to ART is an integral part of the perinatal HIV care continuum from the time of HIV diagnosis through conception, pregnancy, and delivery. Provided virologic suppression is sustained, continual ART from preconception to delivery has the dual benefit of maintaining or improving maternal health while effectively preventing perinatal HIV transmission. To maximize the benefits of ART in WLHIV, regimen selection requires attention to treatment history and viral resistance patterns. Additionally, the risk of teratogenicity of ART must be considered, but limited experience with antiretroviral (ARV) agents during pregnancy can present a unique challenge [1].

Among ARV-naïve adults living with HIV in the United States, HIV-1 integrase strand transfer inhibitors (INSTIs) are the recommended initial regimens for most people or in certain clinical situations. Elvitegravir (EVG) is a commonly prescribed INSTI that requires boosting by cobicistat (COBI) in combination with emtricitabine and tenofovir disoproxil fumarate (STRIBILD, Gilead Sciences, Inc.) or with emtricitabine/tenofovir alafenamide (GENVOYA, Gilead Sciences, Inc.), providing a convenient single-tablet regimen option. However, EVG has very limited data in pregnancy [2–4]. EVG pharmacokinetics during pregnancy in 30 women found lower EVG and COBI levels in the second and third trimesters compared with postpartum. HIV RNA at delivery was <50 copies/mL in 76% of women [5]. Updated perinatal guidelines from November 2017 state, “Elvitegravir/cobicistat is not recommended for initial use in pregnancy until more data are available” [1]. The recommendations further state, “For women who present in pregnancy on elvitegravir/cobicistat, providers should consider switching to more effective, recommended regimens and if elvitegravir/cobicistat is continued, viral load should be monitored frequently, and consider therapeutic drug monitoring if available.”

The rationale for prescribing INSTIs within fixed-dose combination pills taken once daily includes improved efficacy, improved adherence, and rapid decline in viral load [2]. However, there is a knowledge gap on clinical use and maternal viral suppression with EVG during pregnancy. There are theoretical concerns of teratogenicity with INSTIs based on preliminary results from an ongoing observational study in Botswana, which found that pregnant WLHIV who conceived on the INSTI dolutegravir (DTG) gave birth to infants with higher rates of neural tube birth defects (4/426, 0.94%; 95% confidence interval [CI], 0.37%–2.4%) compared with those on non-DTG ARVs at conception (14/11 300, 0.12%; 95% CI, 0.07%–0.21%) [6, 7]. Our multicenter study presents important and timely data on maternal/neonatal outcomes and virologic suppression in pregnant WLHIV receiving EVG-containing ART during pregnancy.

## METHODS

We conducted a retrospective multicenter study of pregnant WLHIV using EVG-containing ART between January 2014 and March 2017 at 9 tertiary care centers in the United States. Women were included if they received EVG at any time during pregnancy. Women with missing delivery data or with elective or spontaneous abortion before 22 weeks were excluded. If a site identified a pregnant woman with HIV on EVG, but there was no corresponding delivery at that site, she was not included due to missing delivery data. Institutional review board approval was completed at every site. Each site reviewed the medical records of all pregnant WLHIV receiving care at their institution during the study dates. Women receiving EVG at any time during pregnancy were identified and included. Demographic data and medical, obstetrical, and neonatal outcomes were collected via chart abstraction form. De-identified data were sent to Emory University for analysis.

Women were categorized into 3 groups: (1) EVG initiated before pregnancy and continued through delivery, (2) EVG initiated during pregnancy, (3) EVG discontinued before delivery. Group 3 included women who were on EVG at conception or initiated EVG during pregnancy but switched to another regimen before delivery. The primary outcome of interest was maternal virologic suppression at delivery, defined as HIV-1 RNA <40 copies/mL. Secondary outcomes included route of delivery, maternal complications, including hypertensive disorders and infection, and obstetrical/neonatal outcomes, including gestational age at delivery, occurrence of birth defects, and neonatal HIV status. These outcomes were analyzed for all pregnancies and also stratified by singleton or twin gestation.

### Statistical Methods

Demographics, maternal medical history, and delivery characteristics were summarized using means and standard deviations, medians and interquartile ranges (IQRs), or frequencies and percentages, as appropriate. Descriptive statistics were presented overall and by EVG group. One-way analysis of variance (ANOVA) and chi-square tests of independence were employed to evaluate bivariate associations between EVG groups and patient characteristics. When continuous data were non-normal or expected frequency counts were low (<5), nonparametric equivalents were used (ie, Kruskal-Wallis and Fisher exact tests). When omnibus tests were significant, all pairwise tests were considered, and significance was reported after adjustment using the adaptive Holm procedure [8]. Unadjusted and adjusted binary logistic regression was employed to evaluate the association between viral load suppression and EVG use groups. Adjusted estimates controlled for maternal race, age, substance abuse, depression/mental illness, low CD4 count (<200), perinatal transmission of HIV, and preterm delivery (gestational age <37 weeks). For all logistic regression results, odds ratios, 95% Wald confidence intervals, and *P* values were reported. Statistical analyses were performed using SAS v9.4 (Cary, NC), and significance was evaluated at the .05 level (2-sided).

## RESULTS

A total of 134 pregnant women from 9 sites across the United States met the eligibility criteria. Table 1 outlines the characteristics of pregnant WLHIV on EVG. The majority of women were black/African American (82.7%), with an average age (range) at delivery of 28.6 (15–44) years. The majority of women were multiparous (77.6%), and 14.2% had a history of preterm delivery. The average age at the time of HIV diagnosis (range) was 20.6 (0–38) years; 14.3% of WLHIV in this cohort contracted HIV through perinatal infection.

**Table 1.** Demographics of Pregnant WLHIV on Elvitegravir by Medication Group

	All (n = 134)	EVG Initiated Before Pregnancy & Continued Through Delivery (Group 1) (n = 68)	EVG Initiated During Pregnancy (Group 2) (n = 52)	EVG Discontinued During Pregnancy (Group 3) (n = 14)	*P* Value	1 vs 2	1 vs 3	2 vs 3	No.
Age at delivery, y	28.0 (24.0–33.0)	28.0 (25.0–33.5)	28.0 (23.8–32.2)	25.0 (21.2–31.5)	.239	0.222	0.136	0.556	133
Race					**.023**	**0.014**	0.589	**0.020**	133
Asian	1 (0.75)	1 (1.47)	0 (0.00)	0 (0.00)					
Black or African American	110 (82.7)	52 (76.5)	48 (94.1)	10 (71.4)					
Hispanic or Latino	6 (4.51)	4 (5.88)	0 (0.00)	2 (14.3)					
Native Hawaiian/ Pacific Islander	1 (0.75)	0 (0.00)	1 (1.96)	0 (0.00)					
White	15 (11.3)	11 (16.2)	2 (3.92)	2 (14.3)					
BMI at first prenatal visit, kg/m^2^	29.6 (23.2–35.8)	31.5 (22.8–36.9)	28.1 (23.7–34.6)	27.5 (22.9–32.6)	.668	0.463	0.488	0.811	132
Parity					**.024**	**0.034**	0.057	0.753	134
Nulliparous	30 (22.4)	9 (13.2)	16 (30.8)	5 (35.7)					
Multiparous	104 (77.6)	59 (86.8)	36 (69.2)	9 (64.3)					
Tobacco use					.758	0.640	0.748	0.638	132
Current	26 (19.7)	11 (16.7)	12 (23.1)	3 (21.4)					
Never	83 (62.9)	43 (65.2)	30 (57.7)	10 (71.4)					
Past	23 (17.4)	12 (18.2)	10 (19.2)	1 (7.14)					
History of PTB	19 (14.2)	11 (16.2)	6 (11.5)	2 (14.3)	.771	0.647	1.000	0.674	134
Comorbidities–hypertension	25 (18.7)	13 (19.1)	9 (17.3)	3 (21.4)	.949	0.987	1.000	0.708	134
Comorbidities–diabetes	1 (0.75)	1 (1.47)	0 (0.00)	0 (0.00)	1.000	1.000	1.000	.	134
Comorbidities–active substance Abuse	25 (18.7)	11 (16.2)	12 (23.1)	2 (14.3)	.654	0.473	1.000	0.716	134
Comorbidities–psychiatric illness	55 (41.0)	32 (47.1)	18 (34.6)	5 (35.7)	.355	0.237	0.630	1.000	134
Start EVG >2nd trimester	8 (5.97)	0 (0.00)	8 (15.4)	0 (0.00)	**.001**	**0.001**		0.187	134
Age at HIV diagnosis, y	22.0 (18.0–27.0)	22.0 (18.0–26.0)	23.0 (17.8–30.0)	19.0 (19.0–23.0)	.407	0.542	0.270	0.223	133
Congenital infection	19 (14.3)	8 (11.8)	9 (17.3)	2 (15.4)	.662	0.549	0.659	1.000	133
HIV diagnosis					**<.001**	**<0.001**	**0.037**	0.198	131
Current pregnancy	23 (17.6)	0 (0.00)	21 (40.4)	2 (15.4)					
Prior pregnancy	28 (21.4)	18 (27.3)	7 (13.5)	3 (23.1)					
Not related to pregnancy	80 (61.1)	48 (72.7)	24 (46.2)	8 (61.5)					

Data are presented as No. (%) or median (interquartile range). Virologic suppression: HIV-1 RNA <40 copies.

Abbreviations: BMI, body mass index; EVG, elvitegravir; PTB, preterm birth; WLHIV, women living with HIV.

Of the 134 women, 45 (33.1%) were not taking ARVs before pregnancy. These women were initiated on ART at a median gestational age (range) of 20 (7–35) weeks. Of the 82 women who were on EVG at the time of conception, 75 (91.5%) were on EVG/cobisistat/emtricitabine and tenofovir disoproxil fumarate, and 7 (8.5%) were on EVG/cobisistat/emtricitabine/tenofovir alafenamide.

Women were divided into 3 groups for comparison (Table 1): (1) EVG initiated before pregnancy and continued through delivery (n = 68, 51.5%), (2) EVG initiated during pregnancy (n = 52, 38.8%), (3) EVG discontinued before delivery (n = 14, 10.4%). Of those not on EVG at delivery, 13 were on EVG at the time of conception and were changed to another ARV regimen during pregnancy, and 1 woman was changed to EVG during pregnancy and changed again before delivery.

### Virologic Suppression

Among 134 pregnant WLHIV who received EVG at any time during pregnancy, viral suppression at delivery occurred in 81.3%. In women who initiated EVG before pregnancy and continued through delivery (group 1), the rate of virologic suppression at delivery was 88.2%. In women who initiated EVG during pregnancy (group 2), the overall rate of suppression was 75.0%. The earlier the EVG was started in the pregnancy, the higher the rate of viral suppression (first trimester: 87.5%; second trimester: 84.6%; third trimester: 37.5%). Of the 8 women who started EVG in the third trimester, 3/8 (37.5%) had viral suppression and 5/8 (62.5%) had an HIV viral load between 41 and 1000 copies/mL at delivery. In women who took EVG during pregnancy but discontinued before delivery (group 3), the rate of viral suppression was 71.4%. Table 2 shows the characteristics and HIV outcomes of our cohort by EVG use in pregnancy.

**Table 2.** HIV Viral Suppression in Pregnant WLHIV on Elvitegravir by Medication Groups

	All (n = 134)	EVG Initiated Before Pregnancy & Continued Through Delivery (Group 1) (n = 68)	EVG Initiated During Pregnancy (Group 2) (n = 52)	EVG Discontinued During Pregnancy (Group 3) (n = 14)	*P* Value	1 vs 2	1 vs 3	2 vs 3	No.
Entered pregnancy on ART	89 (66.9)	67 (100)	9 (17.3)	13 (92.9)	**<.001**	**<0.001**	0.173	**<0.001**	133
CD4 < 200 initial visit	19 (14.3)	8 (11.8)	9 (17.6)	2 (14.3)	.632	0.520	0.677	1.000	133
HIV viral load initial visit, copies/mL					**<.001**	**<0.001**	0.134	**0.017**	134
<40 (undetectable)	66 (49.3)	52 (76.5)	7 (13.5)	7 (50.0)					
41–200	7 (5.22)	2 (2.94)	4 (7.69)	1 (7.14)					
201–1000	7 (5.22)	3 (4.41)	3 (5.77)	1 (7.14)					
>1000	54 (40.3)	11 (16.2)	38 (73.1)	5 (35.7)					
HIV viral load at delivery, copies/mL					.194	0.115	0.217	0.892	134
<40 (undetectable)	109 (81.3)	60 (88.2)	39 (75.0)	10 (71.4)					
41–200	12 (8.96)	4 (5.88)	6 (11.5)	2 (14.3)					
201–1000	1 (0.75)	1 (1.47)	0 (0.00)	0 (0.00)					
>1000	12 (8.96)	3 (4.41)	7 (13.5)	2 (14.3)					
Viral suppression at delivery	109 (81.3)	60 (88.2)	39 (75.0)	10 (71.4)	.094	0.099	0.205	0.744	134

Data are presented as No. (%).

Abbreviations: ART, antiretroviral therapy; EVG, elvitegravir; WLHIV, women living with HIV.

Overall, the 3 groups were demographically similar. Overall virologic suppression at delivery was not statistically different between the 3 groups (*P* = .093). Unadjusted and model-adjusted odds ratios of virologic suppression at delivery by medication use group revealed no significant differences.

### EVG During Pregnancy—Side Effects and Drug Changes

Nausea/vomiting with EVG in pregnancy was reported in 10.3%. Adverse drug effects associated with EVG that were reported in more than 1 patient were upper extremity numbness (n = 3) and difficulty swallowing the pill (n = 2). Women from group 3 (EVG discontinued before delivery, n = 14) were evaluated to determine the reason for EVG discontinuation. The most common reason was physician preference due to lack of safety data on EVG in pregnancy (n = 8). Most of these women were switched to emtricitabine/tenofovir disoproxil fumarate with a boosted protease inhibitor +/- raltegravir or dolutegravir. Two women who conceived on EVG were changed to alternate regimens due to concern for high viral load and drug resistance. Three women discontinued EVG during pregnancy due to reported side effects, including headache and nausea/vomiting.

### Delivery Data and Neonatal Outcomes

Among the 134 pregnancies, there were 140 neonates born, due to 6 pairs of twin pregnancies. Table 3 outlines delivery and neonatal outcomes. The average gestational age at the time of delivery was 37 weeks 6 days. The overall rate of preterm birth was 20.0% (singleton rate: 22/128, 17.2%; twin rate: 6/12, 50.0%). Less than half were delivered by cesarean section (n = 66, 47.5%). The noted indication for cesarean section was repeat in 24.2% and HIV in 19.6%.

**Table 3.** Delivery and Neonatal Outcomes in WLHIV on Elvitegravir During Pregnancy

	All (n = 140)	Singletons (n = 128)	Twins (n = 12)	*P* Value	No.
Delivery gestational age, y	38.2 (37.0–39.1)	38.3 (37.1–39.2)	36.8 (36.2–37.2)	**.001**	140
Preterm birth, <32 wk	5 (3.57)	5 (3.91)	0 (0.00)	1.000	140
Preterm birth, <37 wk	28 (20.0)	22 (17.2)	6 (50.0)	**.015**	140
Route of delivery				**.021**	139
C-section	66 (47.5)	56 (44.1)	10 (83.3)		
Vaginal	73 (52.5)	71 (55.9)	2 (16.7)		
Complications–hypertension	20 (14.3)	20 (15.6)	0 (0.00)	.215	140
Complications–postpartum hemorrhage	4 (2.86)	4 (3.12)	0 (0.00)	1.000	140
Complications–gestational diabetes	3 (2.14)	3 (2.34)	0 (0.00)	1.000	140
Complications–infection	3 (2.14)	3 (2.34)	0 (0.00)	1.000	140
Apgar score <7–1 min	23 (17.3)	20 (16.5)	3 (25.0)	.435	133
Apgar score <7–5 min	6 (4.51)	5 (4.13)	1 (8.33)	.439	133
Low birth weight	26 (19.1)	20 (16.1)	6 (50.0)	**.012**	136
NICU admission	20 (14.5)	18 (14.3)	2 (16.7)	.686	138
IUFD	2 (1.43)	2 (1.56)	0 (0.00)	1.000	140
Birth defect	2 (1.46)	2 (1.60)	0 (0.00)	1.000	137
Neonatal HIV status				1.000	132
Negative	131 (99.2)	121 (99.2)	10 (100)		
Positive	1 (0.76)	1 (0.82)	0 (0.00)		
Neonatal prophylaxis				1.000	110
Zidovudine	100 (90.9)	90 (90.0)	10 (100)		
Zidovudine/nevirapine	6 (5.45)	6 (6.00)	0 (0.00)		
Zidovudine/nevirapine/lamivudine	4 (3.64)	4 (4.00)	0 (0.00)		

Data are presented as No. (%) or median (interquartile range).

Abbreviations: IUFD, intrauterine fetal demise; NICU, neonatal intensive care unit; WLHIV, women living with HIV.

Of the 137 reported neonates, 2 birth defects were detected (rate of 1.5%). One was a case of hydronephorosis in a mother on EVG/cobicistat/emtricitabine/tenofovir disoproxil fumarate before pregnancy and continued throughout. The second was an encephalocele case in a mother who entered pregnancy on tenofovir disoproxyl fumurate/emtricitabine, darunavir, ritonavir, who was changed to atazanavir and elvitegravir/cobicistat/emtricitabine/tenofovir disoproxil fumurate at 9 weeks due to drug side effects. Two intrauterine fetal demises (IUFDs) were identified (1.4%). One IUFD occurred in a woman diagnosed with HIV during the current pregnancy at 31 weeks during admission for hypertensive complications. She was started on elvitegravir/cobicistat/emtricitabine/tenofovir disoproxil fumurate but subsequently developed preeclampsia and had an IUFD at 35 weeks. The other IUFD occurred in a woman diagnosed with HIV 2 years before pregnancy, who was virologically well controlled before and during pregnancy on EVG/cobicistat/emtricitabine tenofovir alafenamide. She had an IUFD at 34 weeks, with placental abruption noted at the time of delivery.

One neonate was diagnosed with HIV infection (positive HIV DNA at birth, 2 months, and 4 months), resulting in a perinatal transmission rate of 0.8%. This transmission occurred in a woman with significant depression and active substance abuse, resulting in 4 antepartum hospitalizations. Her initial viral load was 18 251 copies/mL on lamivudine/zidovudine and lopinavir/ritonavir, and due to nausea/vomiting, she was changed to EVG/cobicistat/emtricitabine tenofovir alafenamide at approximately 15 weeks. Despite this medication change, her viral load at delivery remained elevated, at 13 324 copies/mL. No other neonates were documented to be HIV-infected on completion of study data abstraction. There were no reported neonatal deaths.

## DISCUSSION

This study adds to the very limited data on EVG use during pregnancy. In this multisite cohort, EVG use during pregnancy was well tolerated and associated with viral suppression rates (81.3% overall) comparable to those reported from other cohorts. Notably, women who entered pregnancy on EVG and continued throughout had high rates of viral suppression at delivery (88.2%). The Women and Infant Transmission Study of 630 HIV-infected pregnant women found that only 68% had an undetectable viral load at delivery [9]. A US multicenter observational study found that 86.9% of pregnant WLHIV who initiated ART during pregnancy had undetectable viral loads at delivery, with lower rates of viral suppression at delivery (82.4%) among African American women [10]. Our population was predominately African American. A more recent publication among women in the HIV Outpatient Study (n = 253) from 1996–2015 found that viral suppression was only 60.1% at the time of delivery [11]. Reducing time to HIV viral suppression is critical in pregnancy. A recent study evaluating the outcomes of mother–infant pairs using dolutegravir for HIV treatment during pregnancy found a viral suppression rate of 77.2% in 66 women [12]. A study of time to clinically relevant reduction in HIV RNA in pregnant women using INSTI-containing and non-INSTI-containing ARVs in pregnancy found that INSTIs induced more rapid viral suppression [2]. Although more safety data are needed, the high rates of viral suppression and lack of perinatal transmission among those who were adherent to EVG may support the use of EVG during pregnancy.

Recent pharmacokinetic (PK) data have noted increased rates of subtherapeutic EVG drug levels and reduced levels of its cobicistat booster in the third trimester, raising concerns for viral nonsuppression and increased risk for perinatal HIV transmission [5]. It is unclear if the reduction in drug level noted with EVG in pregnancy is related to PK changes of EVG alone, PK reduction in its cobicistat booster, or both. EVG and cobicistat have been shown to transfer from maternal to fetal circulation via the placenta [13]. The 1 case of perinatal transmission in our cohort occurred from nonadherence, which may have reflected maternal mental illness, and was not likely attributed to pregnancy-related pharmacokinetics of EVG. Consideration has been given to whether higher EVG doses are necessary to reduce the risk of virologic failure and risk of perinatal transmission. However, currently EVG is used as a component of a fixed-dose combination tablet, impeding the ability to make dose adjustments. Given that the rate of HIV transmission in our cohort was not higher than anticipated and that rates of viral suppression were high, dose adjustments may not be needed.

Among our cohort of women exposed to EVG during pregnancy, there was a case of an encephalocele. This pregnancy was formally dated by a 10-week ultrasound, and EVG was initiated at 9 weeks, when the neural tube has closed. The percentage of birth defects observed (1.5%) is similar to that reported in the Antiretroviral Pregnancy Registry and the Centers for Disease Control and Prevention’s population surveillance rate [14, 15]. Given concerns with DTG and the lack of fixed-dose single-INSTI regimens, further large studies of birth outcomes are needed with EVG.

The rate of preterm birth in our cohort was higher than the national rate; however, multiple studies have identified a possible association with ARV use and preterm delivery [16–20]. The perinatal guidelines recommend that clinicians be aware of a possible increased risk of preterm delivery with ARVs; however, given the maternal benefits and reduction in perinatal transmission, ARV medications should not be withheld. Multiple studies have found an increased risk of IUFD in WLHIV on ARVs in pregnancy, with rates ranging from 0.5% to 11.4% [20–26]. The 2 (1.5%) pregnancies that ended in IUFD in our study were complicated by obstetric conditions (placental abruption and maternal hypertension), which likely contributed to fetal demise. Larger studies are also needed to assess if EVG use is associated with preterm delivery and/or stillbirth.

Although this is the largest cohort to date of EVG use in pregnancy, it is still limited by the relatively small sample size and retrospective methodology. Additionally, we did not have a direct comparison of WLHIV on different regimens under care at the same time. The exclusion of elective or spontaneous abortion before 22 weeks could have introduced a potential under-reporting bias in regards to risk of intrauterine fetal demise and birth defects. Nevertheless, this study adds to the evidence regarding the real-world use of EVG during pregnancy and perinatal outcomes.

In conclusion, despite concerns regarding the increased risk of viremia in the second and third trimesters, EVG use in pregnancy was associated with high, sustained, and expected levels of HIV virologic suppression and a low rate of perinatal HIV transmission. As INSTIs are part of treatment for HIV, the number of pregnancies occurring in women on EVG will likely increase. Given the recent World Health Organization/Food and Drug Administration caution on dolutegravir use in pregnancy among reproductive-age women, the number of once-daily fixed-dose combination pills available to this population has become extremely limited. The INSTIs, including EVG, within fixed-dose, single-regimen, once-daily pills improve efficacy, ease of administration, adherence, and tolerability and result in a rapid decline in HIV viral load—all of which are of significant benefit in pregnancy. Although further investigation is necessary, our findings offer support for the overall efficacy and safety of use of EVG-containing ART during pregnancy.
